# An additive effect of leading role in the organization between social participation and dementia onset among Japanese older adults: the AGES cohort study

**DOI:** 10.1186/s12877-017-0688-9

**Published:** 2017-12-29

**Authors:** Yuta Nemoto, Tami Saito, Satoru Kanamori, Taishi Tsuji, Kokoro Shirai, Hiroyuki Kikuchi, Kazushi Maruo, Takashi Arao, Katsunori Kondo

**Affiliations:** 1 Graduate School of Sports Sciences, Waseda University, Saitama, Japan; 2 Department of Social Science, National Center for Geriatrics and Gerontology, Obu City, Aichi Japan; 3 Department of Preventive Medicine and Public Health, Tokyo Medical University, Tokyo, Japan; 4 Human Resource Management Department, ITOCHU Techno-Solutions Corporation, Tokyo, Japan; 5 Center for Preventive Medical Sciences, Chiba University, Chiba, Japan; 6 Department of Human Sciences, School of Law and Letters, University of the Ryukyus, Okinawa, Japan; 7 Department of Clinical Epidemiology, Translational Medical Center, National Center of Neurology and Psychiatry, Tokyo, Japan; 8 Faculty of Sports Sciences, Waseda University, 〒359-1164 Saitama Prefecture, Tokorozawa, Mikajima, 2−579−15, Saitama, Japan; 9 Center for Well-being and Society, Nihon Fukushi University, Mihama, Aichi Japan; 10 Department of Gerontology and Evaluation Study, Center for Gerontology and Social Science, National Center for Geriatrics and Gerontology, Obu City, Aichi Japan

**Keywords:** Japan, Social participation, Leadership role, Dementia onset, Cohort study

## Abstract

**Background:**

Several previous studies reported social participation may reduce the incident of dementia; therefore, the type of positions held in the organization may relate to dementia onset. However, this hypothesis remains largely unknown. The purpose of the present study was to examine the additive effect of a leadership position in the organization on dementia onset and social participation among elderly people in a local community, according to data from a Japanese older adults cohort study.

**Methods:**

Of 29,374 community-dwelling elderly, a total of 15,313 subjects responded to the baseline survey and were followed-up from November 2003 to March 2013. To evaluate the association between dementia onset and social participation as well as the role in the organization, we conducted Cox proportional hazard regression analysis with multiple imputation by age group (aged 75 years older or younger). The dependent variable was dementia onset, which was obtained from long-term care insurance data in Japan; independent variables were social participation and the role in the organization to which they belonged (head, manager, or treasurer). Covariates were sex, age, educational level, marriage status, job status, residence status, alcohol consumption, smoking status, and walking time, instrumental activities of daily living, depression, and medical history.

**Results:**

During the follow-up period, 708 young-old elderly people (7.7%) and 1289 old-old elderly people (27.9%) developed dementia. In young-old elderly, relative to social non-participants, adjusted Hazard Ratio (HR) for dementia onset for participants (regular members + leadership positions) was 0.75 (95% confidence interval (CI), 0.64–0.88). Relative to regular members, adjusted HR for dementia onset for non-participants was 1.22 (95% CI, 1.02–1.46), for leadership positions 0.81 (95% CI, 0.65–0.99). The results for old-old elderly participants did not show that any significantly adjusted HR between dementia onset and social participation, the role in the organization.

**Conclusions:**

In young-old elderly people, social participation might have a positive effect on dementia onset, and holding leadership positions in organization could lead to a decrease in risk of dementia onset by almost 20% than regular members.

**Electronic supplementary material:**

The online version of this article (10.1186/s12877-017-0688-9) contains supplementary material, which is available to authorized users.

## Background

The number of dementia patients has increased dramatically because of the aging population worldwide. In 2010, more than 35 million people developed dementia and it is estimated that increase to 115 million people in 2050 [1]. The population aging rate of Japan is 26.7% in 2015, and prevalence of dementia will increase from 2.8 million (9.5%) in 2010 to 4.7 million (12.8%) in 2025 [2].

Identifying factors related to dementia onset is fundamental for improving preventive strategies; several systematic reviews and meta-analyses have identified some modifiable factors related to cognitive function or dementia onset [3–5], and social participation is one of the factors related to dementia onset [5]. The following, which are promoted by social participation, decrease risk of dementia: increasing physical activity (leaving one’s home), accessing emotional support by expanding social networks, and increasing frequency of cognitive activity by obtaining a social role [6]; however, most of them only focused on absence of social participation and dementia onset or cognitive function, and the additive effect of leadership positions remains largely unknown.

Some observational studies investigated the relationship between leadership positions and health status. According to Ishikawa et al. [7], holding leadership positions on the association was related to a 12% risk reduction of mortality. Takagi et al. [8] suggested that performing leadership positions was significantly related to low odds ratio (OR) for depression for women (OR, 0.57; 95% CI, 0.37–0.88). Having leadership positions within civic groups may decrease the risk of dementia considerably; elderly people who manage the organization to which they belong perform various tasks or acquire roles that stimulate brain function or are beneficial to their health more so than compared with regular members, and this positively affects cognitive function.

The degrees of relationship between social participation and dementia onset may be different according to age group; in old-old (aged 75 or over), the age-related change has a greater effect on physical or mental health than in the young-old (aged 65–74) [9], and social participation can be a burden to the old-old. Therefore, to examine the relationship between social participation and dementia onset by age group is needed.

The purpose of the present study was to assess the additive effect of leadership positions in civic groups on the association between dementia onset and social participation among older adults in a local community, using data from a large cohort study (the Aichi Gerontological Evaluation Study: AGES).

## Methods

### Data

This study was based on data from the Aichi Gerontological Evaluation Study (AGES) project as a part of the Japan Gerontological Evaluation Study (JAGES). JAGES is a largely Japanese prospective cohort study aiming to find out the details of related factors for major health problems among the older adults, such as depression, dementia, or functional deterioration [10, 11].

### Participants

**Fig. 1:**
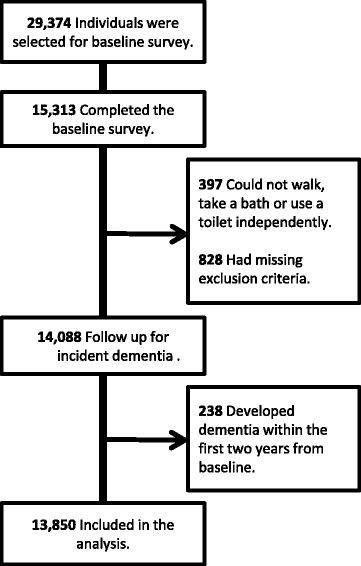
Flow of participants thorough the study

Participants were chosen from within six municipalities in Aichi prefecture, consisting of urban, semi-urban, and rural settings. Out of 49,707 older adults aged 65 years and older in a local community who did not receive public long-term care insurance benefits, 29,374 individuals were selected using two methods: random sampling in two larger municipalities, and a complete survey in four semi-urban or rural municipalities. In October 2003, we conducted the baseline mail survey. A total of 15,313 individuals completed the baseline self-administrated questionnaire and followed up from November 2003 to March 2013. To identify the predictive factors for dementia onset, we involved relatively healthy older adults, and excluded individuals with any premonitory symptoms of dementia, such as being unable to walk, take a bath or use a toilet independently. Individuals who developed dementia within two years of the baseline were also excluded to clarify the relationship between dementia onset and initial conditions (Fig. 1).

The Ethics Committee on Research of Human Subjects at Nihon Fukushi University approved this study protocol.

### Measurements

#### Incident dementia

Dementia onset was determined using disabling dementia, which is defined as incident functional disability with dementia. This was obtained from long-term care insurance data managed by local municipalities, as described previously [12]. Briefly, the degree of functional disability was evaluated according to a two-step procedure: on-site assessment of physical and mental condition by an agent from the home care provider, and further assessment by the Long-term Care Approval Board, consisting of health care professionals (doctors, nurses, caseworkers, or others) that referenced the results of on-site assessment and the primary physician’s report, which is a standard form for assessing medical conditions and physical functions by a home physician [2]. Dementia was determined according to the Degree of Independence in Daily Living for Elderly with Dementia (Dementia Scale) [13, 14]. This scale was developed by the Japanese Ministry of Health, Labour and Welfare, and health professionals in Japan use it to assess physical and cognitive function and classify individuals into levels I–IV and M. Level I means that the individuals have symptoms of dementia, but will be able to maintain an independent daily life. Level II indicates that the individuals show some symptoms and behaviors causing trouble in their daily life or some difficulties with communication, but could continue to live independently if monitored. Level III indicates that the individuals have the same symptoms as Level II patients, but more frequently, and sometimes require care to support their daily lives. Level IV indicates that the individuals have the same symptoms as in Level III, but more frequently, and always need care in their daily lives. Level M indicates individuals with severe mental or physical diseases and behavioral disorders, who require specialized medical care. We defined individuals scoring levels II to IV or M as having dementia. A previous study has shown that the Dementia Scale is well correlated with the Mini Mental State Exam score [15].

#### Social participation and leadership positions in an organization

The scale of social participation was taken from the Japanese General Social Survey [16], and categorized organizations into following eight types: neighborhood association, senior citizen club/fire-fighting team, religious group, political organization or group, industrial or trade association, volunteer group, citizen or consumer group, hobby group, and sports group or club. Participants were asked whether they were members of each association and their frequency of participation; those who answered “I do not participate in any organization” and “participate in the organization” but “very little” for frequency of participation were classified as “non-members”. Therefore, the individuals who belonged to one or more associations were asked their position in the organization; those who serve as head, manager or treasurer were categorized as having “leadership positions”, while others were classified as “regular members”.

#### Covariates

In this study, demographic variables, health behavior, and health status were included as covariates.

Demographic variables consisted of sex, age (65–69, 70–74, 75–79, 80–84, 85 years and over), educational attainment (less than 10 years, 10 or more years), marital status (married, other), residential status (solitary, other), employment (worker, non-worker), health behavior including alcohol status (drinker, non-drinker), smoking status (smoker/ former smoker, never-smoker), walking time (less than 30 min/day, 30 min/day and longer), health status included instrumental ADLs (IADLs) (the subscale of Tokyo Metropolitan Institute of Gerontology Index of Competence: TMIG-IC [17]), medical history (heart disease, stroke, hypertension, diabetes), depression (Geriatric Depression Scale − Short Version, GDS-SV [18]). Those who earned full score for TMIG-IC were categorized as “high”, the GDS-SV cut-off was 5, as in a previous study [19], and subjects who scored above the cut-off were categorized as “depressed”.

### Statistical analysis

To handle missing data, we carried out multiple imputation with full conditional specification, and created 50 multiply imputed datasets [20]. Imputed model included incident of dementia, social participation and leading positions, demographic variables, health behavior, and health status. Therefore, Cox proportional hazard models were used on these datasets. These estimates and their standard errors were combined using Rubin’s rules [21], and Hazard Ratio (HR) or confidence interval (CI) was calculated. For comparison, Cox proportional hazards model was used on the subset of complete case data.

We calculated HRs for incident of dementia according to social participation and by age group (young-old, old-old) using the Cox proportional hazards model to examine the relationship between these factors, and carried out a similar analysis model that exchanged social participation and leading role variables to assess the additive effects of leading positions. We used a level of significance of less than 5% in all analyses. SAS 9.4 (SAS Institute, Cary, NC) was used for all calculations.

## Results

Of 29,374 individuals, 15,313 completed the baseline survey (response rate, 52.1%). Non-responders were younger, and there was no difference between sexes. Of the 15,313 subjects, 13,850 were included in the analysis. A total of 1463 individuals were excluded from analysis; 397 could not walk, take a bath or use a toilet independently, 828 had missing exclusion criteria, and 238 developed dementia within the first two years from baseline (Fig. 1). The mean follow-up period was 7.9 years (standard deviation, 2.4 years), and the number of missing values across each variable varied between 0 (0%) and 933 (10.1%) in young-old, 0 (0%) and 721 (15.6%) in old-old; the total number of individuals who had incomplete data among the all variables was 2629 (28.5%) in young-old and 1663 (36.0%) in old-old. The number of individuals who died during follow-up was 1611 (17.5%) in young-old and 1363 (29.5%) in old-old.

Of the 13,850 subjects of the analysis, 9234 (66.7%) were young-old and 4616 (33.3%) were old-old. Of these young-old, 708 (7.7%) developed dementia, 3003 (32.5%) were non-members, 2514 (27.2%) were regular members, 2784 (30.1%) were in leadership positions, whereas in old-old, 1289 (27.9%) developed dementia, 1774 (38.4%) were non-members, 1289 (27.9%) were regular members, and 832 (18.0%) were in leadership positions (Table 1). Table 2 shows that the incidence of dementia onset increased with age. The incidence in each category of old-old individuals was much higher than in young-old participants.

**Table 1:** Initial characteristics of the participants

		Young-old (*n* = 9234)		Old-old (*n* = 4616)	
		*n*	%	*n*	%
Dementia onset	No-dementia	8526	92.3	3327	72.1
	Dementia	708	7.7	1289	27.9
Sex	Male	4714	51.1	2080	45.1
	Female	4520	48.9	2536	54.9
Social participation	Non-participants	3003	32.5	1774	38.4
	Regular-members	2514	27.2	1289	27.9
	Leadership positions	2784	30.1	832	18.0
	Missing	933	10.1	721	15.6
Age	65–69	5082	55.0	–	–
	70–74	4152	45.0	–	–
	75–79	–	–	2827	61.2
	80–84	–	–	1269	27.5
	≥ 85	–	–	520	11.3
Educational attainment	< 10 yrs	5286	57.2	2849	61.7
	≥ 10 yrs	3896	42.2	1712	37.1
	Missing	52	0.6	55	1.2
Marital status	Married	7343	79.5	2647	57.3
	Single	1766	19.1	1891	41.0
	Missing	125	1.4	78	1.7
Living arrangement	Living with others	8294	89.8	3900	84.5
	Living alone	779	8.4	569	12.3
	Missing	161	1.7	147	3.2
Occupational status	Employed	2806	30.4	608	13.2
	Not employed	6296	68.2	3916	84.8
	Missing	132	1.4	92	2.0
Walking time (per day)	< 30 min	2794	30.3	1586	34.4
	≥ 30 min	5523	59.8	2548	55.2
	Missing	917	9.9	482	10.4
Medical history					
Heart disease	No	8164	88.4	3809	82.5
	Yes	1070	11.6	807	17.5
Stroke	No	9119	98.8	4520	97.9
	Yes	115	1.2	96	2.1
Hypertension	No	6266	67.9	2905	62.9
	Yes	2968	32.1	1711	37.1
Diabetes	No	8168	88.5	4157	90.1
	Yes	1066	11.5	459	9.9
Alcohol consumption	Non-drinker	5535	59.9	3317	71.9
	Drinker	3582	38.8	1181	25.6
	Missing	117	1.3	118	2.6
Smoking	Never smoked	5312	57.5	2800	60.7
	Past smoker/smoker	3615	39.1	1601	34.7
	Missing	307	3.3	215	4.7
Depression	normal	6004	65.0	2591	56.1
	depressed	2304	25.0	1316	28.5
	Missing	926	10.0	709	15.4
IADL	High	7649	82.8	3196	69.2
	Low	1335	14.5	1182	25.6
	Missing	250	2.7	238	5.2

**Table 2:** Incidence rates (1000 person-years) of dementia onset by sex, age, and educational attainment

	Young-old		Old-old	
	Incidence rate	95% CI	Incidence rate	95% CI
Sex				
Male	9.3	8.3–10.4	36.2	32.8–39.9
Female	9.3	8.4–10.5	43.9	40.7–47.4
Age				
65–69	5.5	4.8–6.4	–	–
70–74	14.3	13.0–15.8	–	–
75–79	–	–	31.3	28.8–34.1
80–84	–	–	51.5	46.3–57.3
≥ 85	–	–	83.3	71.8–96.8
Educational attainment				
< 10 yrs	10.2	9.2–11.3	42.6	39.4–45.9
≥ 10 yrs	8.9	7.8–10.1	36.2	32.6–40.1

The results of Cox proportional hazards model on the imputed data indicated that the crude HR for dementia onset for regular members or those holding leadership positions, compared with non-members, was 0.65 (95% CI, 0.55–0.75), and adjusted HR was 0.75 (95%CI, 0.64–0.88) in young-old, whereas crude HR was 0.73 (95% CI, 0.64–0.82), but adjusted HR was non-significant in old-old (Table 3).

**Table 3:** Relationship between social participation and dementia onset

	Crude		Adjusted	
	HR	95% CI	HR	95% CI
Young-old(*n* = 9234)				
Social participation				
Non-participants	reference		reference	
Participants	0.64	0.55–0.75	0.75	0.64–0.88
Old-old(*n* = 4616)				
Social participation				
Non-participants	reference		reference	
Participants	0.73	0.65–0.82	0.91	0.81–1.03

Adjusted for sex, age, educational attainment, marital status, living arrangement, occupational status, walking time, medical history, alcohol consumption, smoking, depression, and IADL

Table 4 shows the relationship between having a leading role and dementia onset. In young-old, both crude HR and adjusted HR for dementia onset for non-members, relative to regular members, were significant (crude HR, 1.38; 95%CI, 1.15–1.65, adjusted HR, 1.22; 95% CI, 1.02–1.46), and crude HR or adjusted HR for leadership positions were also significant (crude HR, 0.76; 95% CI, 0.61–0.94, adjusted HR, 0.76; 95% CI, 0.65–0.99); however, in the old-old group, there was not significant adjusted HR.

**Table 4:** Relationship between having a leadership positions and dementia onset

	Crude		Adjusted	
	HR	95% CI	HR	95% CI
Young-old(*n* = 9234)				
Regular-members	reference		reference	
Non-participants	1.38	1.15–1.64	1.22	1.02–1.46
Leadership positions	0.76	0.61–0.94	0.81	0.65–0.999
Old-old(*n* = 4616)				
Regular-members	reference		reference	
Non-participants	1.30	1.15–1.48	0.99	0.86–1.13
Leadership positions	0.86	0.72–1.02	0.98	0.83–1.14

Adjusted for sex, age, educational attainment, marital status, living arrangement, occupational status, walking time, medical history, alcohol consumption, smoking, depression, and IADL

## Discussion

The present study showed that social activity non-members have a greater risk of incident dementia than social activity members, and members in leadership positions have a significantly lower risk compared with the non-leading members in the young-old group. However, in the old-old group, non-significant differences in dementia risk were observed. These findings seem to suggest that social participation might be effective for prevention of dementia, and this preventive effect could become stronger in the young-old group if leadership positions are taken.

Our findings are broadly consistent with those of previous studies. Kuiper et al. [4] assessed the relationship between social participation and incidence of dementia through meta-analysis. The results of this analysis revealed that individuals with less social participation had a higher risk of dementia onset relative to subjects with higher levels of social participation (RR, 1.41; 95% CI, 1.13–1.75). Although the mechanism underlying the association between social participation and incidence of dementia was not identified, the following pathways were possible: 1) higher level of physical activity due to leaving the home may promote cognitive reserve [6], 2) frequent contact with others may cause positive emotional states such as increased self-esteem, social competence, and adequate mood, which lead to lower stress levels [22], 3) performing various activities (e.g., engaging in a hobby, calculating the scores of games) that stimulate cognitive function serves to prevent a cognitive decline (“use it or lose it” theory) [23]. The present study implies that social participation might have a suppressive effect on the incidence of dementia, but the effect may be different based on participation in social activities. Although the reasons for the additive effect of a leadership role on incidence of dementia are not fully understood, one reason might be the difference in the frequency of social participation. Compared with regular members, individuals who take on leadership roles such as president, facilitator or treasurer have more frequent opportunities for social participation, and also take responsibility for actions to manage group activities (e.g., holding meetings, planning activities, and communicating with regular members). In this study, the proportion of individuals engaging in group activities more than once a month was higher among those in leadership positions than regular members (81.7% vs 64.8%, data not shown). Higher frequency of social participation may help to strengthen the health benefits of social participation [24], or enable individuals to obtain information that supports a healthy lifestyle [25]. Socially-responsible activities may improve the quantity or quality of stimulation of the brain’s cognitive function, or maintain better mental health [8]. However, we did not investigate the type of activity, or use laboratory data, so this is only speculation. As little is known about the mechanism behind the increased positive effect of leadership on cognitive function, further investigation is needed.

In contrast to the young-old group, there were no significant relationships between social participation or leadership and dementia onset in the old-old group. These results support the findings of previous studies [11, 26, 27]. Iwasa et al. [26] suggested that social participation was not attributed to the prevention of cognitive decline among Japanese community-dwelling elderly aged 70 years and over, based on the data from a five-year prospective cohort study. One possible explanation is that as the prevalence of individuals with health problems is much higher in this group than in the young-old group, the relationship between social participation and dementia onset in old-old elderly may be relatively weaker than that in the young-old group. In the present study, health status such as diabetes, depression, and Independent Activity of Daily Living were strongly related to dementia onset (Additional file 1); therefore, these health problems may be the major correlated factors of incidence of dementia in old-old elderly. However, we may have underestimated this relationship in the old-old group for several reasons. First, the percentage of individuals who had died or moved out during follow-up period was much higher (29.5%) among the old-old than young-old (17.5%), which means that about 30% of the old-old participants had died or moved out before developing dementia. Secondly, the presence or absence of social participation and leadership were assessed at baseline, but prior experience was not assessed; therefore, old-old participants who had experienced social participation or leadership before the assessment but had already retired from these activities at the time of baseline assessment were categorized as non-members. Thus, in this study, the category of non-member in the old-old group contained those who were non-members later in life and those who were members before the study period. These reasons can be attributed to underestimation of the association between social participation and incidence of dementia. Further studies of the association of social participation with dementia onset in old-old elderly people are needed.

This study has several limitations. First, the incidence of dementia in this study was obtained from the results of an examination and judgment by the Certification Committee of Needed Long-Term Care in the participant’s municipality. Therefore, underestimation of dementia incidence might have occurred, because every dementia patient does not necessarily submit an application to the Certification Committee. Second, as the type of dementia was not assessed, such as Alzheimer disease, vascular dementia, or Lewy body dementia, the effect of social participation or leadership on each type of dementia remained unclear. Third, the response rate of the baseline survey was 52.1%, meaning that non-responders may have induced selection bias. In this study, the characteristics of non-responders were unknown, but we think it is possible that old-old people or those with lower health status may have been less likely to respond to the survey. There may therefore have been differences in baseline characteristics between study participants and non-participants. Fourth, as the experience of social participation or leadership before the baseline survey was not assessed, the relationship between social participation and dementia onset may be affected by the results of these factors, especially among old-old participants. Future studies evaluating this association should take into account the subject’s experience of social participation and leadership before the baseline survey. Fifth, this study could not identify which types of social activity or leadership were related to the incidence of dementia. Further studies are needed to examine this issue, especially qualitative studies that assess the influence of social participation or a leadership role on older adults’ daily lives. Finally, as the assessment of social participation and leadership were carried out only at the baseline survey, the influence of change in status of participation during the follow-up period on the relationship was not clear.

In summary, despite the above-mentioned limitations, this study revealed that social participation might have a repressive effect on the incidence of dementia and also leadership within the activity group might have stronger positive effect on dementia incidence among young-old adults. These finding should be used to encourage young-old adults to participate in and take leadership positions in social activity organizations.

## Conclusions

In young-old elderly people, social participation might have a positive effect on the prevention of dementia onset, and leadership within a group may lead to a reduction of risk of dementia onset of almost 20%, compared with regular members.

## Additional file

Additional file 1: Relationship between having a leadership positions and dementia onset in old-old. (XLSX 13 kb)
